# Diagnostic Criteria for Problematic Internet Use among U.S. University Students: A Mixed-Methods Evaluation

**DOI:** 10.1371/journal.pone.0145981

**Published:** 2016-01-11

**Authors:** Wen Li, Jennifer E. O’Brien, Susan M. Snyder, Matthew O. Howard

**Affiliations:** 1 School of Social Work, University of North Carolina at Chapel Hill, Chapel Hill, NC, United States of America; 2 School of Social Work, Georgia State University, Atlanta, GA, United States of America; University of Ariel, ISRAEL

## Abstract

Empirical studies have identified increasing rates of problematic Internet use worldwide and a host of related negative consequences. However, researchers disagree as to whether problematic Internet use is a subtype of behavioral addiction. Thus, there are not yet widely accepted and validated diagnostic criteria for problematic Internet use. To address this gap, we used mixed-methods to examine the extent to which signs and symptoms of problematic Internet use mirror DSM-5 diagnostic criteria for substance use disorder, gambling disorder, and Internet gaming disorder. A total of 27 university students, who self-identified as intensive Internet users and who reported Internet-use-associated health and/or psychosocial problems were recruited. Students completed two measures that assess problematic Internet use (Young’s Diagnostic Questionnaire and the Compulsive Internet Use Scale) and participated in focus groups exploring their experiences with problematic Internet use. Results of standardized measures and focus group discussions indicated substantial overlap between students’ experiences of problematic Internet use and the signs and symptoms reflected in the DSM-5 criteria for substance use disorder, gambling disorder, and Internet gaming disorder. These signs and symptoms included: a) use Internet longer than intended, b) preoccupation with the Internet, c) withdrawal symptoms when unable to access the Internet, d) unsuccessful attempts to stop or reduce Internet use, e) craving, f) loss of interest in hobbies or activities other than the Internet, g) excessive Internet use despite the knowledge of related problems, g) use of the Internet to escape or relieve a negative mood, and h) lying about Internet use. Tolerance, withdrawal symptoms, and recurrent Internet use in hazardous situations were uniquely manifested in the context of problematic Internet use. Implications for research and practice are discussed.

## Introduction

Problematic Internet use may lead to serious psychosocial dysfunction [1]. Problematic Internet use is a serious problem for 6% to 11% of Internet users in the United States [2]. Compared to other age groups, college-aged youth and young adults appear to be at greater risk for problematic Internet use given the pervasiveness of Internet access on college campuses and possibly the freedom from parental supervision many college students experience when living away from home for the first time [3–5]. Epidemiological studies indicate that approximately 5% of U.S. university students suffer from problematic Internet use [6–8].

University students with problematic Internet use may exhibit symptoms of impaired physical health (e.g., obesity, sleep disorders) [9, 10], psychological distress (e.g., depression, social anxiety, attention deficit-hyperactivity disorder [ADHD]) [11–14], and behavioral problems (e.g., substance abuse and behavioral addictions, aggression, self-injurious behaviors) [15–17]. Further, students with problematic Internet use often experience more interpersonal problems [18], and worse school and work performance compared to their problematic Internet use-free peers [6].

Although research has documented the prevalence rates and negative consequences of problematic Internet use, the literature does not reflect a consistent conceptualization of problematic Internet use [19–21]. Specifically, it is unclear whether problematic Internet use should be classified as a type of behavioral addiction [21]. Neurobiological studies indicate that problematic Internet use shares many of the same neurobiological underpinnings as substance use disorder [22–25]. Researchers have paralleled the signs and symptoms of problematic Internet use to those of substance use disorder and behavioral addictions including pathological gambling [1, 26–28]. These researchers have argued that characteristics of problematic Internet use include signs and symptoms such as: preoccupation with Internet use; tolerance (i.e., the compulsion to use the Internet for ever increasing amounts of time); repeated but unsuccessful efforts to control, cut back, or stop Internet use; restlessness, irritability, and other signs of withdrawal when unable to use the Internet; having jeopardized or lost a relationship, job, educational or career opportunity because of Internet use; lying to friends, family members, and others to conceal the extent of involvement with the Internet; and using the Internet to escape or palliate dysphoric moods such as depression and anxiety [1, 27, 28]. In addition, some scholars have argued that people are not addicted to Internet; instead, they are addicted to specific activities on the Internet including online gambling, online gaming, online-shopping, and pornography viewing [21]. Internet is an environment or a delivery mechanism [29], and therefore the concept of Internet addiction should refer to pathological and addictive behaviors related to the specific online activities rather than Internet itself.

Many measurement instruments purporting to assess and diagnose problematic Internet use have been developed based on diagnostic criteria for substance use disorder and gambling disorder [1, 26–28, 30–32]. The proposed diagnostic criteria for Internet gaming disorder have been incorporated into Section III of DSM-5 [31]. These instruments assess domains that largely overlap with signs and symptoms of substance use disorder including salience, tolerance, withdrawal symptoms, and use to regulate mood [32]. Although these instruments often demonstrate good psychometric characteristics [27, 32–35], the extent to which the characteristics of problematic Internet use are similar to signs and symptoms of substance use disorder or behavioral addictions remains unclear and controversial [21]. To address this important gap, our research team conducted an exploratory study using mixed-methods (i.e., descriptive and qualitative results) to investigate the degree to which signs and symptoms that are associated with problematic Internet use, as described by university students who self-identified as having problems with Internet overuse, mirror DSM-5 diagnostic criteria for substance use disorder, gambling disorder, and Internet gaming disorder.

## Methods

To obtain detailed descriptions of college students’ experiences with problem Internet use, our team employed exploratory in-depth focus groups. Quantitative data regarding participants’ sociodemographic characteristics and Internet usage patterns were also collected. In addition, two standardized measures were included to assess signs and symptoms that are associated with problematic Internet use. The University of North Carolina-Chapel Hill Institutional Review Board approved the research project.

### Ethics Statement

This study was approved by the University of North Carolina-Chapel Hill Institutional Review Board and performed in accordance with the Declaration of Helsinki. Written consent was obtained from all participants before the focus groups commenced.

### Participants

The study sample was composed of students at a large public university in the southeastern United States. A recruitment email was distributed via the university student listserv to all undergraduate and graduate students once. Eligibility criteria specified that participants (≥ 18 years of age) must spend greater than 25 hours/week on the Internet for non-school or non-work-related purposes, and have experienced one or more physical or psychosocial problems caused by problematic Internet use. Ultimately, 39 students agreed to participate in the focus groups. However, 12 students did not attend scheduled groups. Thus, four groups were held including 27 students. Each student only participated in one focus group discussion. Students were provided with $20 as compensation for their study participation. Participant characteristics are reported in Table 1.

**Table 1:** Sample Characteristics and Internet Use Patterns of 27 College Students Who Report Problematic Internet Use.

Variables	% (N)	Mean (SD)
Age		21.0 (3.6)
Age first accessed the Internet		9.3 (2.7)
Age first recognized having a problem with Internet use		16.2 (4.3)
Gender		
Male	37.0% (10)	
Female	63.0% (17)	
Race		
White	25.9% (7)	
Black	33.3% (9)	
Asian	33.3% (9)	
Latina/Latino	7.4% (2)	
Student status		
Undergraduate	81.5% (22)	
Graduate	18.5% (5)	
*Total YDQ Score		
YDQ > 5	48.1% (13)	
YDQ = 3 or 4	40.7% (11)	
*CIUS Total Score		
CIUS > 21	96.3% (26)	

*Young’s Diagnostic Criteria (YDQ) ≥ 5 indicates Internet addiction. YDQ scores of 3 or 4 = potential IA.

*Compulsive Internet Use Scale (CIUS) ≥ 21 indicates problematic Internet use.

### Focus Group Materials and Measures

Focus group assessment materials consisted of 22 open-ended questions and a set of objective measurement instruments. The group discussion guide was developed and refined by the investigators based on the extant problematic Internet use literature. Major issues explored in the focus groups included patterns, features, and consequences of problematic Internet use, and signs and symptoms of problematic Internet use.

Young’s Diagnostic Questionnaire (YDQ) and the Compulsive Internet Use Scale (CIUS) were employed to assess problematic Internet use. The YDQ consists of 8 questions that assess signs and symptoms of problematic Internet use. Participants answering “yes” to 5 or more questions were identified as having Internet addiction whereas those meeting 3 or 4 criteria were considered to have “potential Internet addiction [1, 36].” The CIUS includes 14 items rated on a 5-point Likert-type scale, ranging from 0 (never) to 4 (very often). The CIUS assesses severity of compulsive Internet use behavior; higher scores indicate greater severity. Prior studies suggest that the CIUS has an internal consistency reliability of ~.90 [30]. A CIUS cutoff score of ≥ 21 for the identification of problematic Internet use has been recommended [37].

### Data Collection

Written consent was obtained from all participants before focus groups commenced. Prior to each focus group, participants completed the YDQ, CIUS, and a brief sociodemographic survey. The surveys were administered to the group members at the beginning of each focus group discussion. Participants were sufficiently separated from each other to ensure that they could not view each other’s responses. The surveys were collected by the researcher once participants completed it. Each focus group lasted approximately one hour. Six to eight participants attended each focus group. Facilitator prompts were used to assure that a wide range of ideas and opinions were represented. A member of the research team conducted each focus group while another member took notes. The presence of multiple observers at group sessions allowed for observer triangulation to improve the reliability and validity of findings emerging from group discussions [38].

### Data Analysis

Audiotapes of focus group sessions were transcribed verbatim. All authors checked for accuracy of the audiotape transcription. A theory-driven coding approach was used to create an initial list of codes [39]. The key research questions, the interview guide, and the signs and symptoms of substance use disorder informed the preliminary coding scheme. Following this coding plan, three research team members thoroughly reviewed each interview transcript to review and revise the codes in the context of the data [39]. Coding discrepancies among the team were resolved through mutual discussion and agreement. Patterns were identified and the analysts implemented constant comparison procedures. In addition, regular debriefing and consultation among research team members helped guard against research bias [38, 40].

## Results

Almost half (48.1%) of the student sample scored five or more on Young’s Diagnostic Questionnaire (YDQ), and therefore scored above the suggested cut-off point for Internet addiction. Another 40.7% scored a three or four on the YDQ, reflecting the suggested cut off for sub-threshold Internet addiction. The internal consistency of the YDQ in this study was .69. Almost the entire sample (96.3%) exceeded the recommended cutoff for problematic Internet use according to the Compulsive Internet Use Scale (CIUS). In this study, the CIUS had an α = .92. Many participants felt their problematic Internet use could validly be described as an addiction. In discussing this, participants likened their Internet use to substance dependence. In the words of one participant, “If you’re addicted to cigarettes, you can’t really go a day without smoking. Probably you can’t go many days without (the) Internet.” In fact, many participants used terms that referred to the signs and symptoms of substance use disorder when discussing their problematic Internet use, including “withdrawal,” “tolerance,” and “craving.” Participant quotes regarding their problematic Internet use are shown in Table 2. These quotes are reflective of the signs and symptoms generally used for substance use disorder and behavioral addictions. These signs and symptoms are: (a) use longer than intended; (b) preoccupation; (c) withdrawal signs/symptoms; (d) tolerance; (e) unsuccessful attempts to stop or reduce Internet use; (f) craving; (g) loss of interest in other hobbies or activities; (h) excessive use despite problems; (i) use the Internet to escape or relieve negative mood; and (j) lying about use.

**Table 2:** Participants’ Quotes about Signs and Symptoms of their Problematic Internet Use and Participants’ Endorsement Rates on Correspondent Items on YDQ and CIUS.

Signs & symptoms	Quotes	YDQ		CIUS Item	
		Specific Item	% (N)	Specific Item	% (N)
Use longer than intended	“I planned to go on the Internet no more than 2 hours one given day, and at most 4. I even set timers on my phone… I added an additional 30 minutes, and the total added up to 6 hours.”	Do you stay on-line longer than originally intended	96.3% (26)	How often do you continue to use the Internet despite your intention to stop?	80.8% (21)
	“At least for me, it’s very impulsive. Before you realize it, you’ve been on Twitter and 45 minutes have gone by and it’s 3 o’clock in the morning.”				
	“There are times where I postpone bed time. You only want to deal with a little thing, but end up [on the Internet] for another hour or two.”				
Preoccupation	“I don’t know what I really want, but I just want to be online.”	Do you feel preoccupied with the Internet?	81.5% (22)	How often do you think of the Internet, even when not online?	29.6% (8)
				How often do you look forward to your next Internet session?	33.3% (9)
	“I’ll get up and check Twitter; or when I get on the bus to the class, I’ll check Twitter; or in class, I’ll check Twitter, and during lunch, I’ll check Twitter; before I go to sleep, I’ll check Twitter.”				
Withdrawal Signs/ Symptoms	“When I can access the entire world and then I can’t, it can be upsetting. I feel frustrated.”	Do you feel restless, moody, depressed, or irritable when attempting to cut down or stop Internet use?	44.5% (12)	How often do you feel restless, frustrated, or irritated when you cannot use the Internet?	44.4% (12)
	“…during my lack of Internet use, I felt kind of irritated sometimes.”				
	“I would feel anxious about feeling being disconnected, like the feeling of missing something.”				
Tolerance	“I think I’m awake for about 18 hours a day, so probably 15 or 16 of those [are spent on the Internet].”	Do you feel the need to use the Internet with increasing amounts of time in order to achieve satisfaction?	55.6% (12)		
	“I think it [my Internet use] can get worse. I mean, I don’t have a smart phone right now, but I’ll probably get one.”				
Unsuccessful attempts to stop or reduce Internet Use	“During the finals, I tried to deactivate my Facebook account, and I would activate it again in no longer than 30 minutes.”	Have you repeatedly made unsuccessful efforts to control, cut back, or stop Internet use?	74.1% (20)	How often have you unsuccessfully tried to spend less time on the Internet?	48.3% (13)
				How often do you find it difficult to stop using the Internet when you are online?	84.6% (22)
	“When I find that the Internet distracts me, I just turn off the router. And it usually works for a period of time, but not for a long time.”				
Craving	“…it becomes a habit that when I wake up in the morning, the first thing I do is to check Facebook, like repeatedly. If you don’t do it, you’ll feel like you miss something.”						
	“It’s really hard for you to focus if you’re on your computer with the Internet. If I don’t have a computer, I’ll be less likely to crave the Internet because then it is not possible [to get on the Internet]. But if the computer is in my backpack, I’m going to reach for it.”						
Loss of interest in other hobbies or activities	“I would go home, and instead of talking to my aunt and cousins, I just sit on the couch, playing on my laptop or my phone. I don’t really socialize with anybody else. So I don’t really talk with anyone.”				How often do you prefer to use the Internet instead of spending time with others (e.g., partner, children, parents, and friends)?	22.2% (6)
	“Well, I mean, definitely, replacing a lot of things that I probably should be doing. Instead of being on the Internet, be outside exercising or doing something…”					
Excessive use despite problems	“I feel like that if not for the Internet, my grades could be 10 times better. I mean instead of listening to the class lecture, I am on Twitter; instead of doing my homework, I am on Twitter; and I rush my homework to get on Twitter.”	Have you jeopardized or risked the loss of a significant relationship, job, educational, or career opportunity because of your Internet use?	33.3% (9)	How often do you neglect your daily obligations (work, school, or family life) because you prefer to go on the Internet?	44.4% (12)
	“My partner and I complain to each other about the time we spend on the Internet. But I decided we have no choice.”			How often are you short of sleep because of the Internet?	62.9% (17)
	“The biggest thing for me is texting while I’m driving. I have to really work on not doing that. I haven’t suffered any consequences, but the potential consequences always, like, bring me back to the reality that I could die or you know, you can get fined.”			How often do you rush through your homework in order to go on the Internet?	38.5% (10)
Use the Internet to escape or relieve a negative mood	“When I am sad, I go online to watch TV shows, and it just makes me forget about the sad things.”	Do you use the Internet as a way of escaping from problems or of relieving a dysphoric mood (e.g., feelings of helplessness, guilt, anxiety, and depression)?	63.0% (11)	How often do you to on the Internet when you are feeling down?	50% (13)
	“I think the Internet is my number one skip for negative feelings.”			How often do you use the Internet to escape from your sorrows or get relief from negative feelings?	42.3% (11)
	“If I am really depressed- I won’t get on Facebook, I don’t want to talk to anyone, I won’t use anything like a social networking kind of thing. But I’ll definitely go on something like Tumblr to look at funny things for, like, an hour.”				
Lying about use	“If this is something that’s a real problem, then you act like you have a drug problem. Like sometimes I’ll be sitting in front of the computer, and someone asks ‘what are you doing?’ I’m really on the Internet, but I’ll be like ‘I’m just trying to write that paper!’ But I know what I am doing.”	Have you lied to family members, a therapist, or others to conceal the extent of your involvement with the Internet?	25.9% (7)		

### Use Longer than Intended

Taking a substance in larger amounts or over longer periods than was intended is a sign of substance use disorder [31]. This sign is not assessed by DSM-5 criteria for gambling disorder and Internet gaming disorder [31]. Focus group participants reported similar signs in relation to their Internet use. Many group members had experienced being on the Internet longer than they had initially intended. Participants noted that they often lost track of time while on the Internet for recreational purposes, resulting in loss of sleep, less social interaction, and reduced academic work productivity (see Table 2 for direct quotes from focus group participants and participants’ endorsement rates on the correspondent items on the YDQ and CIUS). YDQ results showed that 96.3% of participants reported having stayed on the Internet longer than they intended. Similarly, 80.8% of participants reported that they often/very often continued to use the Internet despite their intention to stop.

### Preoccupation

Preoccupation is characterized by individuals devoting a great deal of time to obtaining and using and/or recovering from the effects of substances [31]. This criterion is also used to assess gambling and Internet gaming disorder in DSM-5 [31], and refers to persistent thoughts of previous gambling/gaming activity, anticipating and planning the next gambling/gaming venture, and thinking of ways to get money for gambling. Preoccupation refers to when substance use, gambling, or Internet gaming has become the dominant activity in an individual’s daily life. Focus group participants reported similar signs with respect to their problematic Internet use. Participants noted spending substantial amounts of time thinking about activities on the Internet, not only while using the Internet but also when not using or anticipating the next session of use (see Table 2 for direct quotes from focus group participants and participants’ endorsement rates on the correspondent items on the YDQ and CIUS). YDQ results showed that 81.5% of participants felt preoccupied with the Internet. According to CIUS, 29.6% of participants frequently thought of the Internet (even when not online), and 33.3% of participants often/very often reported looking forward to their next Internet session.

### Withdrawal Signs/Symptoms

Withdrawal refers to a characteristic syndrome of signs and symptoms that follow abstinence from a substance in a person dependent on that substance [31]. Withdrawal signs and symptoms assessed in the DSM-5 diagnostic criteria for gambling and Internet gaming disorder only include psychological dependence [31]. Such psychological dependence is characterized by feeling restless, irritable, or sad when attempting to cut down or stop gambling or gaming, or when one cannot access games. Similarly, focus group participants reported experiencing psychological withdrawal symptoms when unable to use the Internet. Participants noted negative mood states such as “frustration,” “irritation,” and “anxiety” when they were unable to access the Internet, or had attempted to reduce or stop their Internet use (see Table 2 for direct quotes from focus group participants and participants’ endorsement rates on the correspondent items on the YDQ and CIUS). YDQ indicated that 44.5% of participants had experienced feeling restless, moody, depressed, or irritable when attempting to cut down or stop their Internet use. CIUS results showed that 44.4% of participants frequently experienced feeling restless, frustrated, or irritated when they could not use the Internet.

### Tolerance

Tolerance is characterized by individuals needing increasing amounts of a substance over time to achieve intoxication or desired effects [31]. Tolerance is also a criterion included in the DSM-5 criteria for gambling and Internet gaming disorder [31]. It parallels the criterion for substance use disorder and refers to needs to gamble with increasing amounts of money, or to spend increasing amounts of time engaged in Internet gaming in order to achieve the desired excitement. Focus group participants reported using the Internet in greater amounts due to its accessibility. Some participants noted using the Internet for the entire time they are awake. Participants also indicated that they could use the Internet more when they have smartphones with unlimited data (see Table 2 for direct quotes from focus group participants and participants’ endorsement rates on the correspondent items on the YDQ). YDQ results showed that 55.6% of participants reported feeling the need to use the Internet for increasing amounts of time in order to achieve satisfaction. Tolerance was not examined on the CIUS.

### Unsuccessful Attempts to Stop or Reduce Internet Use

This sign is characterized by individuals having made unsuccessful efforts to stop or cut back on use of a substance [31]. This criterion is also assessed in DSM-5 criteria for gambling and Internet gaming disorder. It refers to a desire to stop or cut back on pathological gambling or gaming behaviors, but being unable to do it [31]. Focus group participants reported similar signs in relation to their Internet use. Participants noted a desire to reduce their Internet use, followed by unsuccessful attempts to stop or reduce their Internet use (see Table 2 for direct quotes from focus group participants and participants’ endorsement rates on the correspondent items on the YDQ and CIUS). YDQ results showed that the majority of participants had repeatedly made unsuccessful efforts to control, cut back, or stop Internet use (74.1%). CIUS results suggested that more than 80% of participants (84.6%) frequently found it difficult to stop using the Internet once they were online, and almost half of the participants (48.3%) reported they had frequently unsuccessfully tried to spend less time on the Internet.

### Craving

Craving refers to strong desires or urges to use a substance [31]. However, craving for gambling or playing Internet games is not assessed in DSM-5 criteria for gambling and Internet gaming disorder. Focus group participants reported craving in regard to their Internet use. Participants noted urges or a strong desire to engage in activities on the Internet, specifically, when Internet access is available to them (see Table 2 for direct quotes from focus group participants). Neither the YDQ nor the CIUS included items related to craving.

### Loss of Interest in Other Hobbies or Activities

A loss of interest in other hobbies or activities is a sign of substance use disorder and Internet gaming disorder [31]. This criteria is not included in the DSM-5 criteria for gambling disorder. Participants noted having lost interest in (or having participated less often in) activities they had previously found enjoyable including “socializing with friends or family,” “going-out,” and “exercising” due to Internet use (see Table 2 for direct quotes from focus group participants and participants’ endorsement rates on the correspondent items on the YDQ and CIUS). CIUS responses indicated that 22.2% of participants often/very often preferred to use the Internet instead of spending time with others. The YDQ did not include questions regarding loss of interest in other hobbies or activities.

### Excessive Use despite Problems

This sign is characterized by continued use of a substance despite a persistent physical or psychological problem associated with substance use, or playing video games on the Internet [31]. This criteria is not included in the DSM-5 criteria for gambling disorder. Focus group participants reported similar behavior vis-a-vis their problem Internet use. Participants noted continued excessive Internet use despite problems such as academic under-achievement, conflict with others about Internet overuse, negative physical outcomes (e.g., inadequate amount of sleep), and Internet use while driving (see Table 2 for direct quotes from focus group participants and participants’ endorsement rates on the correspondent items on the YDQ and CIUS). Another related criterion in the DSM-5 criteria for substance use, gambling and Internet gaming disorder assesses the actual negative consequences (e.g., jeopardizing or actually losing important relationships or work/educational opportunities) of substance use, pathological gambling or Internet gaming. Focus group participants reported adverse health and/or psychosocial consequences due to their problematic Internet use behaviors. The negative consequences related to problematic Internet use have been reported in the previous study by the authors [4]. YDQ results showed that 33.3% of participants had jeopardized or risked the loss of a significant relationship, job, educational, or career opportunity because of their Internet use. CIUS responses indicated that 62.9% of participants frequently experienced being short of sleep because of their excessive Internet use, 38.5% frequently rushed through their homework in order to get on the Internet, and 44.4% frequently neglected their daily obligations because they preferred to access the Internet.

### Use of the Internet to Escape or Relieve a Negative Mood

This sign is characterized by individuals using a substance to cope with negative moods such as depression, guilt, or anxiety; or gambling or playing Internet games when feeling distressed (e.g., helpless, guilty, anxious, or depressed) [31]. However, this criterion is not included in the DSM-5 criteria for substance use disorder. Focus group participants noted engaging in excessive Internet use to escape from, or cope with, negative moods or feelings such as “sadness,” “annoyance,” or “boredom” (see Table 2 for direct quotes from focus group participants and participants’ endorsement rates on the correspondent items on the YDQ and CIUS). YDQ results showed that 63.0% of participants had used the Internet as a way to escape from problems or relieve a dysphoric mood. CIUS findings indicated that half (50.0%) of participants often/very often used the Internet when they were feeling “down,” and almost half (42.3%) frequently used the Internet to escape from their sorrows or get relief from negative feelings.

### Lying about Use

Individuals having lied to family members, a therapist, or others to conceal the extent of their involvement with gambling or gaming on the Internet characterize this theme [31]. However, this criterion is not included in the DSM-5 criteria for substance use disorder. Focus group participants also reported deceit in association with their Internet use. Some participants noted having lied to cover up the extent of their Internet use, such as the amount of time spent on the Internet or the specific activities performed online (see Table 2 for direct quotes from focus group participants and participants’ endorsement rates on the correspondent item on the YDQ). YDQ results showed that 25.9% of participants had lied to family members, a therapist, or others to conceal the extent of their involvement with the Internet. The CIUS did not ask questions regarding this theme.

## Discussion

This study explored the extent to which problematic Internet use behaviors described by university students mirror the DSM-5 criteria for substance use disorder, gambling disorder, and Internet gaming disorder. Overall, signs and symptoms associated with problematic Internet use described by students in this study were similar to those of substance use disorder, gambling disorder, and Internet gaming disorder [31]. Importantly, participant quotes provided detailed descriptions of signs and symptoms of problematic Internet use, and contextualized the related quantitative findings. Although clearly, more rigorous studies are needed, our findings supported the previous evidence suggesting that problematic Internet use could be a type of behavioral addiction [1–2,20,22]. In a recently published qualitative study using the same sample [4], the authors explored the natural history of problematic Internet use; common affective, interpersonal, and situational triggers of Internet overuse; patterns of Internet use; and negative consequence of problematic Internet use among university students. The findings from the recent published study suggested that students’ self-reports of problematic Internet use were consistent with results of standardized measure which were developed based on diagnostic criteria for substance use disorder and gambling disorder, including Young’s Diagnostic Criteria and Compulsive Internet Use Scale [4]. Building upon previous work, the findings of this study further suggest that the signs and symptoms “volunteered” by university students who self-identified as having problems with Internet overuse mirrored signs and symptoms of substance use disorder, gambling disorder, and Internet gaming disorder assessed by DSM-5 criteria.

In line with extant literature, results indicated that there was overlap between the signs and symptoms of problematic Internet use and substance use disorder as well as behavioral addictions [2, 27, 28]; however, the specific context and symptom manifestation for problematic Internet use was distinct. Specifically, the Internet is widely available on college campuses and is normalized within college life. Students can use the Internet anytime and anywhere due to the wide adoption of campus-wide Internet access, smartphones, and data coverage [4]. This may limit the visible manifestation of symptoms such as tolerance in that there is a “ceiling effect” [41]—it is impossible for students to spend *more* than 24 hours in one day on the Internet. In addition, students in the focus groups appeared to be able to access the Internet whenever they have a desire to engage in Internet-related activities. Therefore students in this study may not notice the development of tolerance symptoms. Although more than half (55.6%) of participants responded positively to the YDQ item that assesses tolerance, very few students in the focus group reported using the Internet increasingly to achieve the same level of satisfaction. Measurement of such a construct among populations with problematic Internet use may require researchers and service providers to develop new and distinct diagnostic criteria. Tolerance refers to feeling the need to use the Internet longer periods of time to experience the same amount of satisfaction. Tolerance may also involve the need for more exciting activities on the Internet. When assessing tolerance among students, other information regarding the history and current patterns of Internet use may also need to be assessed in addition to the YDQ item “a need to increasee the amount of time spent on the Internet to achieve same amount of satisfaction.”

Similarly, withdrawal symptoms may also manifest uniquely among populations with problematic Internet use behaviors. While substance use withdrawal generally includes physical symptoms [31], students in this study did not report physiological symptoms of withdrawal. Instead, students reported a myriad of psychological withdrawal symptoms including negative mood states (e.g., depression), and anxiety symptoms (e.g., restlessness). These psychological symptoms have been linked to other substance, gambling, and Internet gaming disorder [1–2, 27,28, 31], and therefore may need to be more closely examined by researchers and service providers when assessing for problematic Internet use among college-aged youth. Further, the results suggest that the “withdrawal like symptoms” could partially related to interpersonal conflict due to Internet overuse or problematic Internet use. Therefore, withdrawal symptoms associated with problematic Internet use should be distinguished from psychological distress that arise in response to other related factors, including stop using the Internet because of conflicts with parents or significant others regarding problematic Internet use.

Finally, Internet use while driving emerged as a key finding under the theme “Excessive Use Despite Problems.” Students reported using the Internet on their phones/tablets to chat with friends, play games, and post on social media sites while driving, despite knowledge that such behaviors are life threatening and illegal in all states. These behaviors strongly mirror substance use behaviors such as drinking and driving. However, as with tolerance and withdrawal symptoms, the context of Internet use while driving is often normalized among young people and so the serious repercussions are minimized by the youth themselves and the service providers who work with them [42].

These findings have implications for future research. Specifically, studies using larger and more representative sample and rigorous designs are needed to further investigate the extent to which the characteristics of problematic Internet use mirror the signs and symptoms of substance use and behavioral addictions. Further studies need to be conducted to closely examine the extent to which the existing instruments and diagnostic criteria adequately assess the domains of problematic Internet use, and to establish the validity of the cutoff points of existing instruments that measure problematic Internet use behaviors. Without validated and sensitive/specific instruments and diagnostic criteria, it is difficult to reliably distinguish pathological from normal Internet users.

In addition, Internet is an environment or a medium, and the addiction or the addictive behavior would pertain to the corresponding activities on the Internet and not to the Internet itself [21]. Thus, the further studies need to focus on the addiction-like symptoms that are associated with specific problematic behaviors related to Internet-use (e.g., Internet gaming disorder, online-gambling, online-shopping, and online pornography viewing). Specific criteria should be developed to assess subtypes of problematic Internet use (such as compulsive pornography viewing and Internet gaming addiction), because each subtype may have different signs, symptoms, and adverse effects.

Such knowledge would be especially important for service providers working with college-aged youth suffering from problematic Internet use and its associated psychosocial problems. While the Internet is necessary for academic work, job performance, and socialization, problematic Internet use has serious adverse effects on psychosocial well-being. Thus, service providers should screen and assess for problematic Internet use among at-risk college-aged youth. Our findings show that tolerance may not be a sensitive criterion; instead, other signs and symptoms, such as psychological withdrawal symptoms, craving, and excessive use despite problems should be included when assessing problematic Internet use.

Study limitations include the small sample size, single site location of the investigation, and exploratory nature of the findings. These factors all may limit the generalizability of results. However, the university where this research was conducted is similar to many other large public universities and the sample was diverse with respect to ethnicity and gender. Further, focus group discussion rather than individual assessment may lead to social desirability biases and influence the validity of the qualitative findings. The data collected were rich and informed by quantitative and qualitative assessments. Perhaps most importantly, given the paucity of research on U.S. college students with problematic Internet use, we hope our findings will stimulate further investigation in this important emerging area.

## Supporting Information

S1 Document Survey Questions for Sociodemographic and Characteristics of Problematic Internet Use, and Focus Group Discussion Guidance. (DOCX)

S2 Document Codebook. (DOCX)

S1 Table Data Set for Sample Characteristics of 27 Participants Who Self-Reported Intensive Internet Use. (DOCX)

S2 Table Data Set for Young’s Diagnostic Questionnaire (N = 27). (DOCX)

S3 Table Data set for the Compulsive Internet Use Scale (N = 27). (DOCX)
