# Supplementary material for: Diagnostic Criteria for Problematic Internet Use among U.S. University Students: A Mixed-Methods Evaluation
Source: PLoS One. 2016 Jan 11;11(1):e0145981. doi: 10.1371/journal.pone.0145981 (PMC4709169; doi:10.1371/journal.pone.0145981)
Supplement: S2 Document — (DOCX) [file pone.0145981.s002.docx]

| Code | Description |
| --- | --- |
| Use longer than intended | Having been on the Internet for non-school- and/or non-work-related activities longer than initially intended or planned. |
| Preoccupation | Having been thinking about previous activities on the Internet and/or anticipating the next session of Internet use. Internet has become the dominant activity in an individual’s daily life. |
| Withdrawal signs/symptoms | Having felt irritable, restless, sad, upset or other psychological discomfort when Internet is taken way or when cannot access Internet. |
| Tolerance | Having felt the need to spend increasing amounts of time on the Internet for non-school- and/or non-work-related activities. |
| Unsuccessful attempts to stop or reduce Internet use | Having repeatedly made unsuccessful efforts to control, cut back, or stop certain activities on the Internet or Internet overuse. |
| Craving | Having had urges or an intensive desire to engaging in non-school- and/or non-work-related activities on the Internet. |
| Loss of Interest in other hobbies or activities | Having lost interest in previous hobbies and entertainment as a results of non-school and/or non-work-related Internet overuse. |
| Excessive use despite problems | Continuing excessive use of Internet for non-school- and/or non-work-related activities despite knowledge of psychosocial problems (e.g., poor school performance, and interpersonal relationship conflicts due to Internet overuse). |
| Use of the Internet to escape or relieve a negative mood | Having used Internet to escape or relieve a negative mood (e.g., feelings of loneliness, sadness, guilt, anxiety, and helplessness). |
| Lying about use | Having lied to family members, therapists, or others regarding the amount of time spent on the Internet for non-school- and/or non-work-related activities and/or the specific activities on the Internet. |
| * The code book is developed based on signs and symptoms that are associated with substance use disorder, gambling disorder, and Internet gaming disorder assessed in DSM-5 criteria. | |

**Document S2 Codebook.**
