# Supplementary material for: Diagnostic Criteria for Problematic Internet Use among U.S. University Students: A Mixed-Methods Evaluation
Source: PLoS One. 2016 Jan 11;11(1):e0145981. doi: 10.1371/journal.pone.0145981 (PMC4709169; doi:10.1371/journal.pone.0145981)
Supplement: S2 Table — (DOCX) [file pone.0145981.s004.docx]

**S2** **Table.** Data Set for Young’s Diagnostic Questionnaire (N = 27)

| Case ID | YDQ Score | Item1 | Item2 | Item3 | Item4 | Item5 | Item6 | Item7 | Item8 |
| --- | --- | --- | --- | --- | --- | --- | --- | --- | --- |
| 1 | 4 | 1 | 1 | 1 | 0 | 1 | 0 | 0 | 0 |
| 2 | 5 | 1 | 0 | 1 | 0 | 1 | 1 | 0 | 1 |
| 3 | 6 | 1 | 1 | 1 | 1 | 1 | 0 | 0 | 1 |
| 4 | 4 | 1 | 1 | 1 | 0 | 1 | 0 | 0 | 0 |
| 5 | 7 | 1 | 1 | 1 | 1 | 1 | 0 | 1 | 1 |
| 6 | 4 | 1 | 0 | 1 | 0 | 1 | 0 | 0 | 1 |
| 7 | 7 | 1 | 1 | 1 | 1 | 1 | 0 | 1 | 1 |
| 8 | 8 | 1 | 1 | 1 | 1 | 1 | 1 | 1 | 1 |
| 9 | 6 | 1 | 1 | 1 | 0 | 1 | 1 | 0 | 1 |
| 10 | 5 | 1 | 0 | 1 | 1 | 1 | 1 | 0 | 0 |
| 11 | 8 | 1 | 1 | 1 | 1 | 1 | 1 | 1 | 1 |
| 12 | 6 | 1 | 0 | 1 | 1 | 1 | 1 | 0 | 1 |
| 13 | 4 | 0 | 0 | 1 | 1 | 1 | 0 | 0 | 1 |
| 14 | 5 | 1 | 1 | 0 | 0 | 1 | 0 | 1 | 1 |
| 15 | 4 | 1 | 1 | 1 | 0 | 1 | 0 | 0 | 0 |
| 16 | 0 | 0 | 0 | 0 | 0 | 0 | 0 | 0 | 0 |
| 17 | 3 | 0 | 0 | 1 | 0 | 1 | 0 | 0 | 1 |
| 18 | 2 | 0 | 0 | 1 | 0 | 1 | 0 | 0 | 0 |
| 19 | 2 | 1 | 0 | 0 | 0 | 1 | 0 | 0 | 0 |
| 20 | 4 | 1 | 1 | 0 | 0 | 1 | 0 | 1 | 0 |
| 21 | 4 | 0 | 1 | 1 | 0 | 1 | 1 | 0 | 0 |
| 22 | 4 | 1 | 0 | 0 | 1 | 1 | 0 | 0 | 1 |
| 23 | 7 | 1 | 1 | 1 | 1 | 1 | 1 | 1 | 0 |
| 24 | 7 | 1 | 1 | 1 | 1 | 1 | 1 | 0 | 1 |
| 25 | 3 | 1 | 0 | 0 | 0 | 1 | 0 | 0 | 1 |
| 26 | 6 | 1 | 1 | 1 | 1 | 1 | 0 | 0 | 1 |
| 27 | 3 | 1 | 0 | 0 | 0 | 1 | 0 | 0 | 1 |
